# Using genomic resources for linkage analysis in *Peromyscus* with an application for characterizing *Dominant Spot*

**DOI:** 10.1186/s12864-020-06969-1

**Published:** 2020-09-11

**Authors:** Zhenhua Shang, David J. Horovitz, Ronald H. McKenzie, Jessica L. Keisler, Michael R. Felder, Shannon W. Davis

**Affiliations:** Department of Biological Sciences, University of South Carolina, Columbia, SC 29208 USA

**Keywords:** *Peromyscus*, Neural crest, *Sox10*, Genomic, DNA polymorphism

## Abstract

**Background:**

*Peromyscus* are the most common mammalian species in North America and are widely used in both laboratory and field studies. The deer mouse, *P. maniculatus* and the old-field mouse, *P. polionotus*, are closely related and can generate viable and fertile hybrid offspring. The ability to generate hybrid offspring, coupled with developing genomic resources, enables researchers to conduct linkage analysis studies to identify genomic loci associated with specific traits.

**Results:**

We used available genomic data to identify DNA polymorphisms between *P. maniculatus* and *P. polionotus* and used the polymorphic data to identify the range of genetic complexity that underlies physiological and behavioral differences between the species, including cholesterol metabolism and genes associated with autism. In addition, we used the polymorphic data to conduct a candidate gene linkage analysis for the *Dominant spot* trait and determined that *Dominant spot* is linked to a region of chromosome 20 that contains a strong candidate gene, *Sox10*. During the linkage analysis, we found that the spot size varied quantitively in affected *Peromyscus* based on genetic background.

**Conclusions:**

The expanding genomic resources for *Peromyscus* facilitate their use in linkage analysis studies, enabling the identification of loci associated with specific traits. More specifically, we have linked a coat color spotting phenotype, *Dominant spot*, with *Sox10*, a member the neural crest gene regulatory network, and that there are likely two genetic modifiers that interact with *Dominant spot*. These results establish *Peromyscus* as a model system for identifying new alleles of the neural crest gene regulatory network.

## Background

*Peromyscus* are the most common and widely distributed mammalian species in North America and includes the deer mouse (*P. maniculatus*), the old-field mouse (*P. polionotus*), and the white-footed mouse (*P. leucopos*) [1]. *Peromyscus* have similar characteristics to old world mice, including the house mouse, *Mus musculus*, which is the most widely used model system for biomedical research. However, *Peromyscus* and *Mus* are more distantly related than their appearance suggests, sharing a last common ancestor 25 million years ago [2]. *Peromyscus* are more closely related to hamsters than to *Mus*.

*Mus* is an exceptional model system for biomedical research, having a wide range of genetic resources, including a fully sequenced and annotated genome, various inbred strains, and the ability to make specific genetic modifications. However, in developing *Mus* laboratory strains, human selection resulted in a genome with contributions from three different *Mus* species, and the process of inbreeding resulted in a lack of genetic diversity [3]. As a result, genome wide association studies (GWAS) using *Mus* typically produce linkage to genomic intervals that are too large to facilitate candidate gene identification [4]. Furthermore, the lack of genetic diversity has reduced the pool of traits that could be identified and eventually characterized in laboratory mice. Recognizing the limitations of inbred *Mus* lines for GWAS and quantitative trait locus (QTL) mapping studies, the Collaborative Cross was initiated to generate genomic variation by combining eight inbred lines and three wild subspecies of *Mus* [5, 6]. By intercrossing the Collaborative Cross strains, a Diversity Outbred stock was generated to reduce the linkage disequilibrium blocks characteristic of the founder inbred lines [6, 7]. The Diversity Outbred stock has been used to map numerous quantitative traits observed in the founding inbred lines, including serum cholesterol levels and heart size [6, 8, 9].

Another option is to use the variation observed in existing outbred stocks to generate smaller linked intervals that facilitate gene discovery. For instance, a GWAS study conducted on outbred *Mus* stocks for variability in high density lipoprotein and hypercholesterolemia produced a linked interval of less than 100 kb, which lead to the identification of allelic differences in *Apoa2* associated with hypercholesterolemia [10]. The key to generating small linked intervals is the frequency of genetic variation. In inbred strains, the lack of genetic variation generates long syntenic regions that result in large blocks of linkage disequilibrium. *Peromyscus* bred in captivity are susceptible to inbreeding depression; therefore, the *Peromyscus* Genetic Stock Center (PGSC) at the University of South Carolina (UofSC) maintains outbred stocks of *P. maniculatus* (BW), *P. polionotus* (PO), and *P. leucopus* (LL). These stocks are maintained by breeding individuals that do not have a common grandparent [11]. Because these outbred stocks have not undergone intentional selection, they maintain both genetic and phenotypic diversity, similar to natural populations [1, 12]. These genetically diverse laboratory stocks provide another animal model with physiological and behavior variation that differs from the Diversity Outbred stocks and other outbred *Mus* stocks, providing an opportunity to study how genetic variation may predispose individuals to disease [13, 14]. Additional genetic variation can be generated in *Peromyscus* because *P. maniculatus* and *P. polionotus* can produce viable and fertile hybrid offspring [1].

*P. maniculatus* and *P. polionotus* are species with distinct behavioral and metabolic differences [1]. The ability to generate hybrids between two *Peromsycus* outbred stocks enables researchers to use QTL analysis to identify genetic loci that underlie the quantitative traits, behavior, or metabolic differences between the two species. For instance*, P. polionotus* build better nests than *P. maniculatus* [15]. A QTL associated with nest building is located on chromosome 4, and the vasopressin gene is located within the QTL. There are no non-synonymous changes in the coding region of vasopressin between the two species; however, *P. maniculatus* express 2.8-fold more vasopressin mRNA in the hypothalamus than *P. polionotus*. This species-specific expression difference results in the species-specific nest building behaviors, as increasing vasopressin in *P. polionotus* inhibits nest building behavior [15]. Other QTL analyses utilizing *P. maniculatus* and *P. polionotus* have identified genetic loci associated with coat color differences and burrowing behavior [16, 17].

There are many behavioral differences between *P. maniculatus* and *P. polionotus* that are associated with human health. For instance, The BW stock of *P. maniculatus* has a high incidence of repetitive behavior, while the PO stock of *P. polionotus* has no repetitive behavior [18, 19]. In humans, repetitive behavior is observed in people with autism and obsessive-compulsive disorder [20]. A QTL analysis for repetitive behavior using *P. maniculatus* and *P. polionotus* hybrids could identify the genetic loci associated with this behavior and provide an animal model for understanding complex behaviors associated with human disease.

The genomic tools that facilitate QTL analysis in *Peromyscus* are actively being developed. The first genomic assembly containing almost 31,000 scaffolds of *P. maniculatus* was released in 2013 (GenBank assembly GCA_000500345.1). Raw whole genome sequencing reads for *P. polionotus* were deposited in the Sequencing Read Archive (SRA) in July 2015 (SRX179420, SRX179421, and SRX179422). An improved, chromosome level assembly for *P. maniculatus* followed in November of 2018 (GenBank assembly GCA_003704035.1), along with chromosome level assemblies of *P. polionotus* (GenBank assembly GCA_003704135.2) and *P. leucopus* (GenBank assembly GCA_004664715.1), in April of 2019 [21]. These resources facilitate the design of linkage mapping studies by providing species specific allelic information across the genome. Restriction site-associated DNA sequencing, RADseq, and its variant double digest RADseq, ddRADseq, facilitate linkage mapping studies by providing high through-put genome wide allelic information, and have been instrumental in several *Peromyscus* QTL studies [15, 16, 22, 23]. While a reference genome is not necessary for RADseq experiments, it facilitates these experiments by providing a scaffold on which to map allelic information.

The continued development of *Peromyscus* genomic resources is required to facilitate linkage analysis studies in *Peromyscus*. We performed a comparative analysis of the *P. maniculatus* and *P. polionotus* genomes, using available genomic datasets, to identify polymorphisms between the two species that can be used for linkage analysis. We used the polymorphic data to design a candidate gene linkage mapping study to identify a genetic locus associated with the *Dominant spot* trait in *P. maniculatus*. This information was also used to identify genetic differences associated with known physiological and behavioral differences between the two species.

## Results

### Analysis of genomic variations between *P. maniculatus* and *P. polionotus*

The ability to generate fertile and viable hybrid offspring from *P. maniculatus* and *P. polionotus* provides an opportunity to identify the genetic loci associated with phenotypic traits that differ between the two species. Capitalizing on this rich genetic resource requires identifying the allelic differences between the two species. We sought to identify allelic differences between *P. maniculatus* and *P. polionotus*, using publicly available genomic resources. The first assembly of the *P. maniculatus* genome was used as the reference genome, and the *P. polionotus* sequencing reads deposited in the SRA database were mapped against the *P. maniculatus* reference genome. For polymorphism determination we required 10 independent *P. polionotus* sequencing reads to be mapped to a specific *P. maniculatus* genomic location (data file available at https://osf.io/4eypx/). With these criteria we determined that 81.9% of the *P. maniculatus* genome was covered by 10 *P. polionotus* sequencing reads, resulting in 38,166,334 polymorphisms between *P. maniculatus* and *P. polionotus*. Among these variations are 34,084,607 single nucleotide polymorphisms (SNPs) and 4,081,727 insertions or deletions (INDELs) between BW and PO (Table 1). Lowering the read mapping requirement to five sequencing reads increased the number of identified polymorphisms to 40,815,360. However, we chose to keep the more stringent criteria for subsequent analysis, recognizing that we are likely underestimating the number of polymorphisms between the species. Using the more stringent 38,166,334 polymorphisms results in a variant rate of one variation every 68 base pairs. We used SnpEff to annotate the potential functional effects of the polymorphisms (data file available at https://osf.io/4eypx/), recognizing that individual variant locations can have multiple annotations, which increases the number of annotations above the total number of variants. More than 23 million variants occur in intergenic regions, with more than 7 million occurring within 5 kb upstream or downstream of an annotated gene, potential impacting transcriptional regulatory sites (Table 1). More than 45 million variants occur in an annotated transcript region, including > 769,000 in annotated exons and > 44 million in annotated introns (Table 1). Within exons, the majority occur in 3′ untranslated regions (UTR).

**Table 1:** Characterization of variants

Variant Type	Count
SNP	34,084,607
Insertion	2,038,754
Deletion	2,042,973
Transcript	45,591,302
EXON	769,092
5′ UTR	96,746
3′ UTR	490,685
INTRON	44,630,345
Splice Acceptor	1215
Splice Donor	1327
Splicing Region	87,301
Intergenic	23,148,376
5 kb Upstream	7,302,504
5 kb Downstream	7,337,171

We characterized the SNPs that occur between *P. maniculatus* and *P. polionotus* for changes in predicted protein coding sequences and found that there are 11,588 genes that contain a coding sequence SNP. Of these variants, 70.2% are silent, 29.6% are missense, and 0.2% are nonsense variants. We identified a set of 10,405 genes that contain a nonsynonymous change, which might result in a functional difference in the protein between the two species. Using the annotated *Mus* genome as a reference, we conducted a gene ontology (GO) term analysis on the list of 10,405 genes with missense or nonsense variants to determine if any biological processes or molecular functions are over or underrepresented within this list of genes. There are 2060 biological processes that are over-represented, and 63 that are under-represented (Table 2 and Additional Files 1 and 2). The processes with the most statistically significant enrichment are typically broad in GO terminology, including cellular process, cellular metabolic process, and metabolic process. *P. maniculatus* and *P. polionotus* are known to have distinct physiological differences, including an almost four-fold increase in blood cholesterol levels in *P. polionotus* compared to *P. maniculatus* and a two-fold increase in blood triglyceride levels in *P. polionotus* over *P. maniculatus* [24]. We searched the list of overrepresented GO terms for terms with a possible relationship with cholesterol and triglycerides and found that the GO terms cholesterol metabolic process, cholesterol homeostasis, cholesterol transport, triglyceride metabolic process, triglyceride biosynthetic process, and triglyceride catabolic process are enriched in this list of genes with potential functional protein changes (*p* = 3.90 × 10^− 6^, 3.05 × 10^− 5^, 3.86 X10^− 4^, 9.86 × 10^− 6^, and 1.81 × 10^− 5^, respectively) (Additional File 1). Each of these GO terms contains from 20 to 77 different genes with nonsynonymous changes, suggesting that there is substantial genetic complexity that may underlie the metabolic differences between the two species. In contrast, there are 63 GO terms that are under-represented in the list, including sensory perception of smell, sensory perception of chemical stimulus, sensory perception, G protein-coupled receptor signaling pathway, and nervous system process, suggesting that these biological processes are more conserved between the two species.

**Table 2:** Top 20 over and underrepresented GO terms in the list of *Peromyscus* genes containing a nonsynonymous SNP between *P. maniculatus* and *P. polionotus*

**Overrepresented GO terms**	**GO term ID**	***p*-value**	**Number of** ***Mus*** **genes with GO term**	**Expected number of genes**	**Number of** ***Peromyscus*** **Genes with non-synonymous SNP**
cellular process	GO:0009987	2.03E-298	15,748	6516	7781
cellular metabolic process	GO:0044237	8.06E-257	9331	3861	5119
metabolic process	GO:0008152	2.96E-243	10,435	4318	5557
primary metabolic process	GO:0044238	5.83E-235	9405	3891	5096
organic substance metabolic process	GO:0071704	8.98E-225	9966	4124	5310
nitrogen compound metabolic process	GO:0006807	2.89E-214	8892	3679	4819
cellular component organization or biogenesis	GO:0071840	5.56E-214	6225	2576	3618
cellular component organization	GO:0016043	9.53E-201	6053	2505	3505
localization	GO:0051179	3.18E-193	5918	2449	3423
cellular macromolecule metabolic process	GO:0044260	2.50E-174	7080	2929	3905
macromolecule metabolic process	GO:0043170	3.99E-170	8461	3501	4506
organonitrogen compound metabolic process	GO:1901564	9.62E-137	6215	2572	3402
developmental process	GO:0032502	4.23E-136	6193	2562	3390
anatomical structure development	GO:0048856	1.40E-130	5810	2404	3198
establishment of localization	GO:0051234	1.11E-127	4518	1869	2588
biological regulation	GO:0065007	1.61E-120	12,092	5003	5874
multicellular organism development	GO:0007275	1.36E-119	5304	2195	2931
transport	GO:0006810	4.07E-118	4378	1811	2494
organelle organization	GO:0006996	2.00E-117	3549	1468	2095
macromolecule modification	GO:0043412	7.62E-114	3623	1499	2121
**Underrepresented GO terms**	**GO term ID**	***p*-value**	**Number of** ***Mus*** **genes with GO term**	**Expected number of genes**	**Number of BW Genes with non-synonymous SNP**
sensory perception of smell	GO:0007608	3.37E-238	1138	471	17
sensory perception of chemical stimulus	GO:0007606	2.58E-228	1236	511	39
sensory perception	GO:0007600	1.17E-116	1784	738	303
G protein-coupled receptor signaling pathway	GO:0007186	2.04E-83	1921	795	410
nervous system process	GO:0050877	8.09E-55	2286	946	606
system process	GO:0003008	2.91E-25	2923	1209	955
phagocytosis, recognition	GO:0006910	3.11E-23	151	62	9
response to pheromone	GO:0019236	1.07E-21	105	43	2
complement activation, classical pathway	GO:0006958	1.28E-19	165	68	16
humoral immune response mediated by circulating immunoglobulin	GO:0002455	3.89E-17	180	74	23
complement activation	GO:0006956	1.02E-15	187	77	27
humoral immune response	GO:0006959	6.06E-15	355	147	78
phagocytosis, engulfment	GO:0006911	4.56E-12	189	78	34
protein activation cascade	GO:0072376	1.11E-11	200	83	38
B cell receptor signaling pathway	GO:0050853	2.16E-11	181	75	33
xenobiotic metabolic process	GO:0006805	2.19E-11	112	46	14
plasma membrane invagination	GO:0099024	6.36E-11	198	82	39
membrane invagination	GO:0010324	1.60E-10	205	85	42
response to leukemia inhibitory factor	GO:1990823	1.06E-09	311	129	78
cellular response to leukemia inhibitory factor	GO:1990830	1.06E-09	311	129	78

The *P. maniculatus* laboratory stock, BW, is known to have a significant incidence of repetitive, or stereotactic, behavior, including repetitive jumping [18, 25, 26]. The *P. polionotus* laboratory stock, PO, does not display stereotactic behaviors. In humans, repetitive movements are associated with autism, obsessive-compulsive disorder, and other neurologic disorders, including tics and schizophrenia [20, 27]. BW animals have been used to study how neuroactive drugs and environmental enrichment can reduce repetitive behavior [28, 29]. BW animals are also less social than PO animals, another hallmark of autism in humans [18, 30]. Our expectation is that there are genetic loci that underlie the stereotactic and social behaviors and we sought to determine if there is genetic variation between BW and PO that may be associated with these behavioral differences. We examined the list of overrepresented GO terms for processes that may be related with autism associated behaviors and found that locomotory behavior and social behavior are both enriched GO terms (*p* = 1.94 × 10^− 5^ and 6.79 × 10^− 4^, respectively) (Additional File 1). In addition, we selected a list of candidate genes that all have a high confidence of being associated with autism in humans from the Simons Foundation Autism Research Initiative (SFARI) database (Table 3). We then identified sequence variations that occur between *P. maniculatus* and *P. polionotus* in this list of autism candidate genes. Each gene analyzed has multiple sequence variations between the two species that could result in a functional change to the protein, including missense changes, nonsense changes, in-frame deletions, and nucleotide variations in splicing regions. In addition, there are numerous differences in untranslated regions, introns, and upstream and downstream sequences that could result in differences in transcript or protein levels, if they occur in regulatory regions.

**Table 3:** Polymorphism characterization in *Peromyscus* genes associated with autism in humans

Gene	SFARI Score	Upstream	5′ UTR	Silent	Missense	In-frame deletion	Splice region	Intronic	3′ UTR	Downstream
*Arid1b*	1S	205		30	6		4	5881	13	
*Adnp*	1S	75		4	3			175	24	
*Ash1l*	1S	205		16	12		3	1089	9	53
*Chd8*	1S	155	2	26			11	663	11	42
*Dyrk1a*	1S	84		4			1	888	19	82
*Kmt2a*	1S	100	2	39	15	1	5	896	19	
*Tbr1*	1	89		2			1	83	1	

**Fig. 1:**
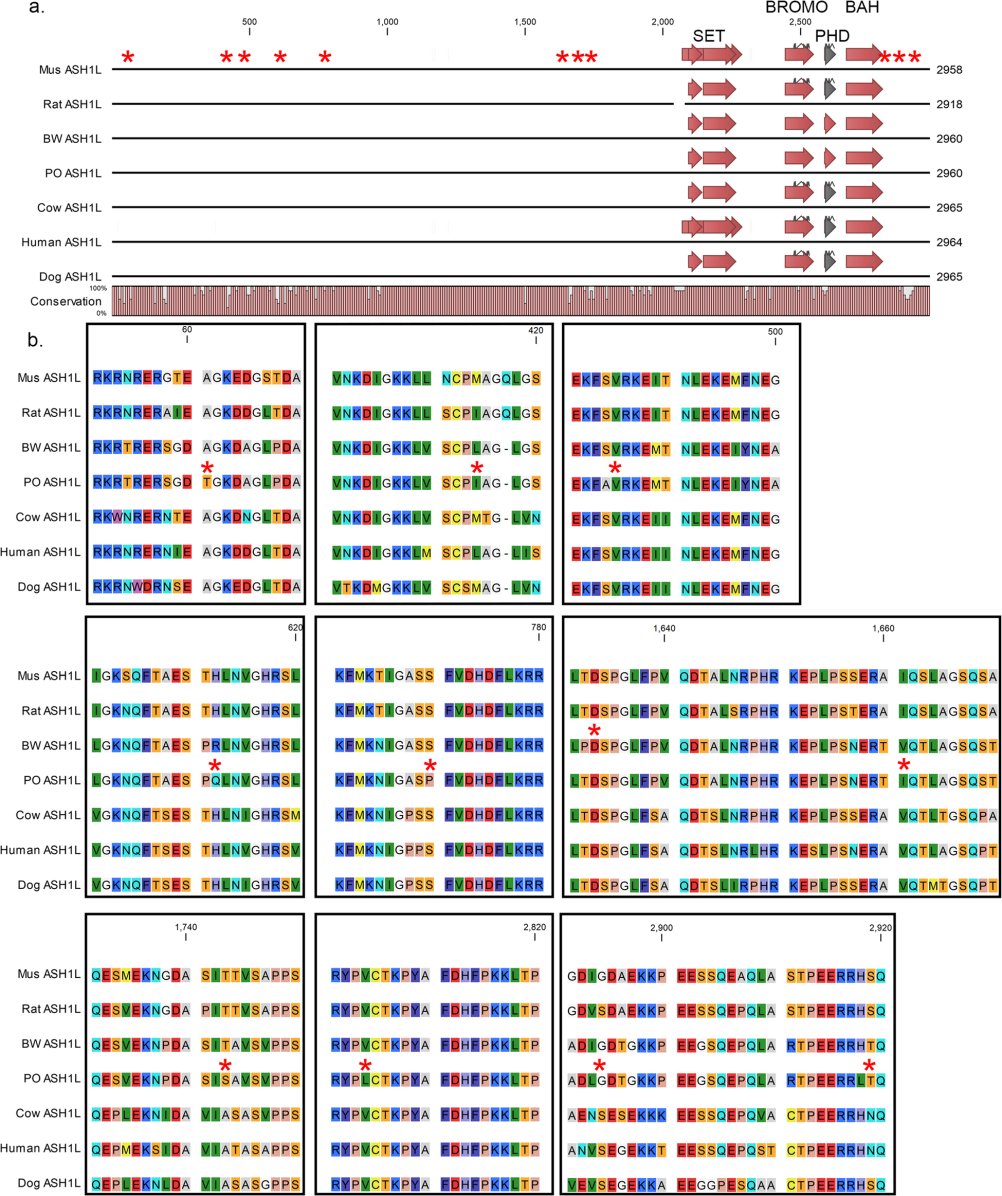
ASH1L protein conservation. **a** Schematic representation of a protein alignment between *Mus*, rat, BW, PO, cow, human, and dog ASH1L proteins. Numbers across the top represent approximate amino acid locations. Block arrows represent conserved protein domains. Conservation graph at the bottom indicates amino acid percent conservation between the seven species. Asterisks indicate approximate locations for amino acid differences between BW and PO. **b** Regional protein alignment for ASH1L in areas surrounding an amino acid difference between BW and PO, with amino acid locations indicated across the top

We wanted to further explore how polymorphisms between BW and PO might result in functional changes by examining polymorphisms in one autism candidate gene, recognizing that there are no identified connections between there autism candidate genes and the behavioral differences between BW and PO. ASH1L is a chromatin modifying protein that is associated with transcriptional activation [31]. De novo mutations in ASH1L have been identified in multiple people with autism, while rarely occurring in controls [32–34]. We aligned select mammalian ASH1L protein sequences to identify highly conserved amino acids and determine if *Peromyscus* amino acid substitutions occur in these conserved residues (Fig. 1). None of the nonsynonymous changes between *P. maniculatus* and *P. polionotus* ASH1L are in the ASH1L conserved protein domains, SET, BROMO, PHD, and BAH, and generally occur in regions with less conservation between mammalian ASH1L proteins. However, at positions 61, 484, 770, 1632, and 2814 there are amino acid substitutions in one *Peromyscus* species where the amino acid is conserved in the other mammals. The S484A, S770P, and T1632P substitutions are intriguing as they remove potential phosphorylation sites in one of the *Peromyscus* species. The potential functional impact of these amino acid substitutions on ASH1L function will require further characterization.

**Fig. 2:**
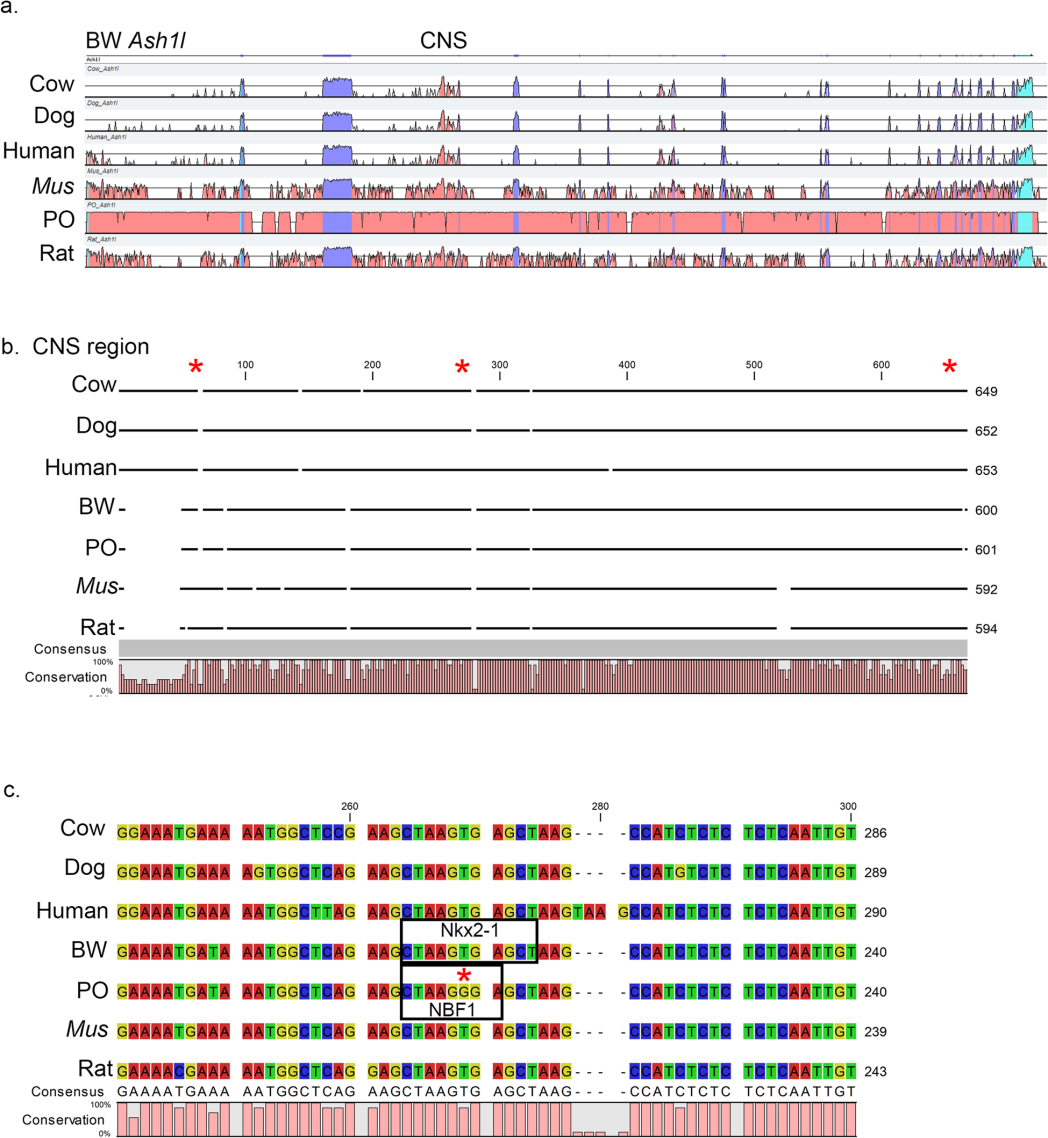
ASH1L nucleotide conservation. **a** VISTA plot for *Ash1l* genomic loci for cow, dog, human, *Mus*, PO, and rat compared to BW *Ash1l*. Blue regions indicate exons and red regions indicate conserved non-coding regions. CNS indicates a conserved non-coding sequence in intron three across all mammals analyzed. **b** DNA sequence alignment for the CNS, indicated in **a** for the seven mammalian species. Numbers across the top represent nucleotide locations. Conservation graph at the bottom indicates percent nucleotide conservation between the seven mammalian species. Asterisks indicate approximate locations for nucleotide differences between BW and PO. **c** Regional nucleotide alignment within the CNS. Asterisk indicates a single nucleotide polymorphism between BW and PO. Boxed regions indicate transcription factor binding sites in BW for NKX2.1 and in PO for NBF1

We also examined possible transcriptional regulatory changes between *P. maniculatus* and *P. polionotus* for ASH1L by generating a VISTA plot to identify conserved non-coding sequences (CNS) in the ASH1L locus [35]. A conserved non-coding sequence occurs in intron 3 of ASH1L in 100 vertebrate species (UCSC Genome Brower: Human GRCh38/hg38 chromosome 1: 155,459,751-155,478,012) and is also found in BW and PO (Fig. 2) [36]. Within this CNS there are three SNPs between *P. maniculatus* and *P. polionotus*. Two of the three *Peromyscus* SNPs are in positions that are not conserved between a group of seven mammalian species. However, one SNP occurs in a region of 16 nucleotides that are completely conserved within the selection of mammalian species (Fig. 2c). We used PROMO to identify potential transcription factor binding sites within this region and found that in six mammalian species, including *P. maniculatus*, the conserved sequences contain a potential NKX2–1 binding site [37, 38]. However, in *P. polionotus* the SNP removes the NKX2–1 binding site and generates a potential EBF1 binding site.

### Restriction enzyme recognition sites in *P. maniculatus*

A QTL analysis or GWAS using *Peromyscus* is likely to utilize RADseq. RADseq is a flexible approach to genomic analysis, as the choice of restriction enzyme used to digest the genomic DNA can be varied to customize the number of sequenced sites, known as RAD markers, across the genome [39]. The number of RAD markers generated is twice the number of restriction enzyme recognition sites. An enzyme that cuts more frequently will generate more RAD markers and, therefore, provide more allelic information than an enzyme that cuts less frequently. We used the *P. maniculatus* reference genome to determine the number of cuts sites and the average fragment size for the enzymes listed in Table 4. This data provides a range of restriction enzymes with recognition sites from approximately 1000 bp apart (DraI) to approximately 1 million bp apart (AscI), enabling an informed choice for restriction enzyme selection in *P. maniculatus* RADseq projects. RADseq generates about 400 bp of sequence information flanking a restriction enzyme recognition site. Because a sequence variant between *P. maniculatus* and *P. polionotus* occurs approximately every 68 base pairs, it is likely that RADseq analysis on F_1_ hybrids will generate informative allelic information at most RAD markers.

**Table 4:** Number of restriction enzyme recognition sites and average fragment size for *P. maniculatus*

Restriction Enzyme	CpG	Number of Recognition sites in ***Peromyscus***	Average fragment size in ***Peromyscus***
ApaI	Yes	384,617	6431
AscI	Yes	2460	1,005,504
AvrII	No	482,638	5125
BamHI	No	410,386	6027
BspQI	No	457,484	5406
BssHII	Yes	115,555	21,405
DraI	No	2,508,985	986
EagI	Yes	47,415	52,167
EcoRI	No	701,369	3527
FseI	Yes	13,191	187,517
HindIII	No	809,283	3056
NaeI	Yes	105,993	23,336
NarI	Yes	89,075	27,769
NheI	No	377,464	6553
NotI	Yes	5842	423,406
PacI	No	146,207	16,918
PmeI	No	34,572	71,547
RsrII	Yes	10,111	244,638
SacI	No	608,101	4067
SacII	Yes	47,658	51,901
SaII	Yes	33,458	73,930
Sbfl	No	78,368	31,563
SgrAI	Yes	13,574	182,226
SmaI	Yes	200,696	12,324
SpeI	No	370,097	6683
SphI	No	654,548	3779
SspI	No	1,559,294	1586
SwaI	No	166,068	14,894
XbaI	No	748,330	3305
XhoI	Yes	109,564	22,576

### Linkage analysis of dominant spot

**Fig. 3:**
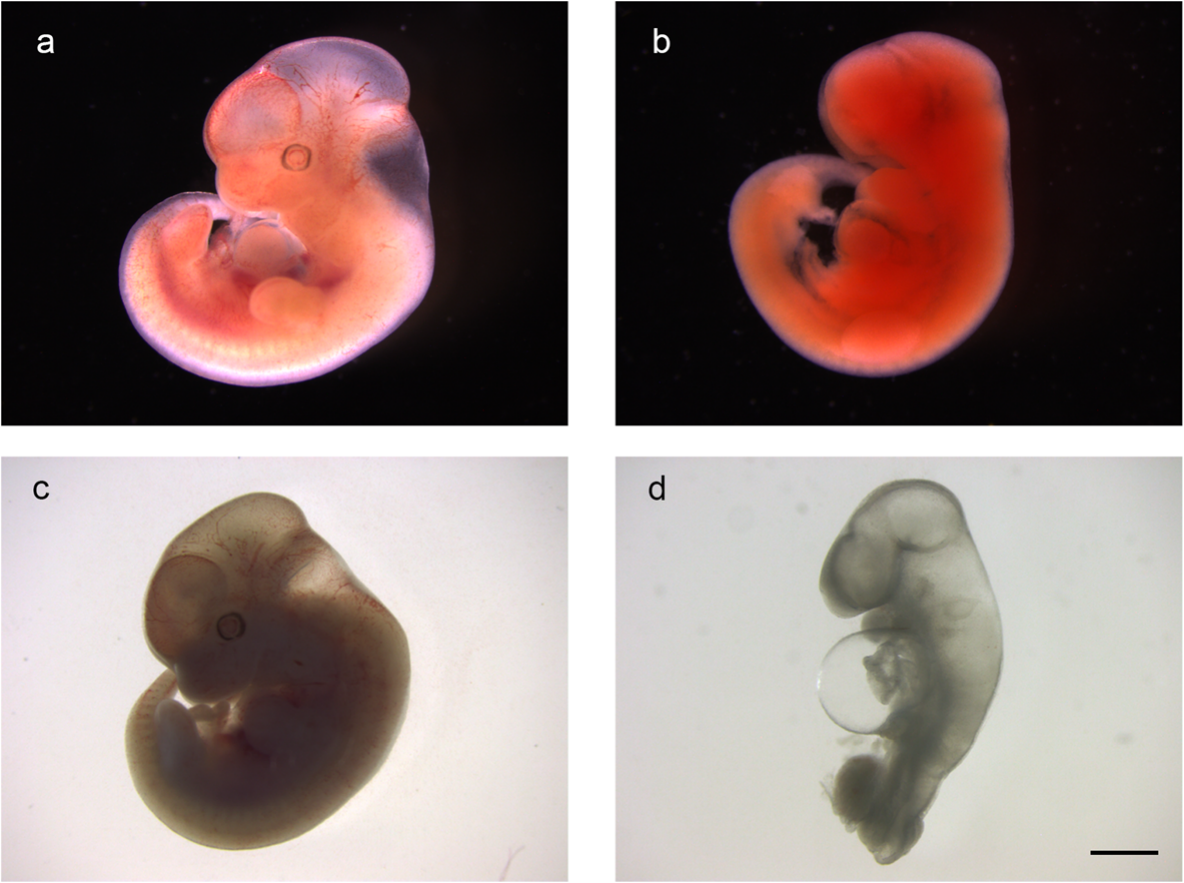
Embryonic lethality of BW *S/S* embryos. **a** Wild type e14.5 embryo. **b** resorbing e14.5 embryo. **c** Wild type e13.5 embryo. **d** e13.5 embryo with malformations, including pericardial swelling and caudal regression. Scale bar equals 1 mm for all images. Images were acquired by the authors

*Dominant spot* is a spontaneous mutation that arose within a wild population of *P. maniculatus* near Morrison, Illinois [1, 40]. The *Dominant spot* trait (*S*) is maintained as heterozygotes on the BW laboratory stock of *P. maniculatus* at the PGSC. PGSC breeding records suggest that *S/S* homozygotes are likely embryonic lethal. We crossed BW *S/+* adults and generated timed pregnancies and observed resorbing embryos at embryonic day of development 14.5 (e14.5), and approximately one quarter of e13.5 embryos have a variable phenotype that includes morphological defects consistent with embryonic lethality (Fig. 3).

**Fig. 4:**
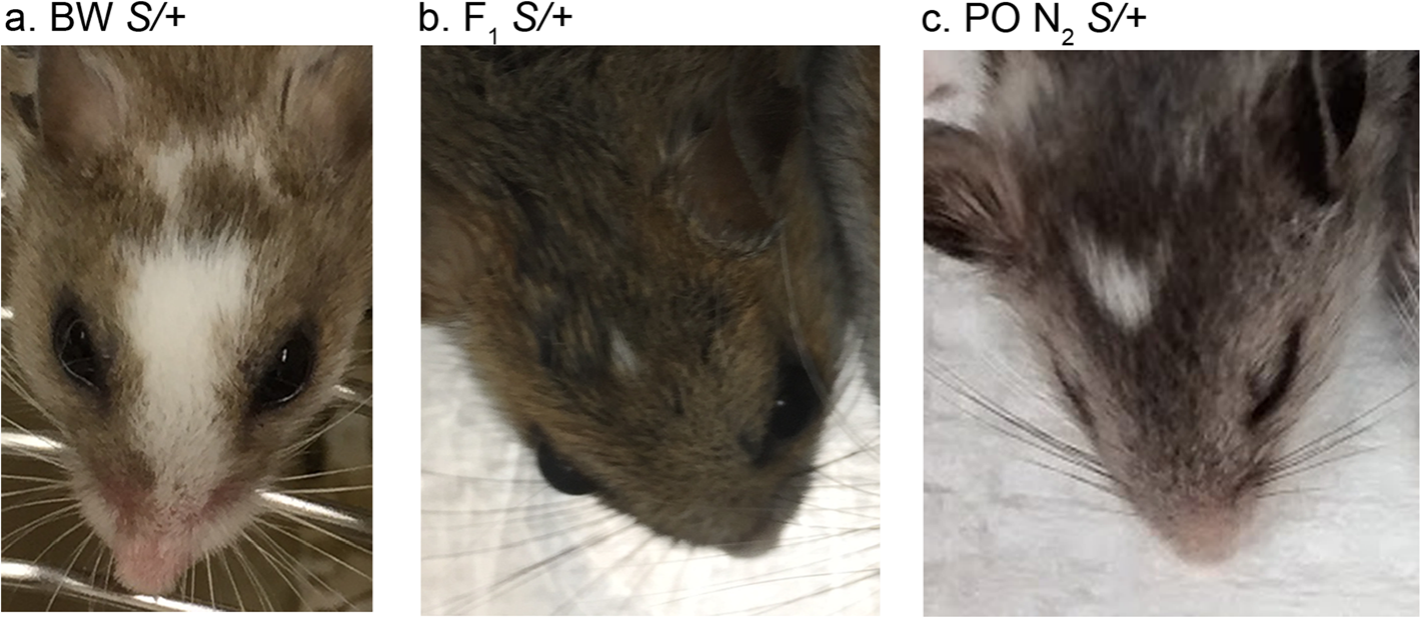
Variation in spot size for the *Dominant spot* trait. **a** Large spot size on an *S/+ P. maniculatus*. **b** Small spot size on an *S/+* F_1_ hybrid. **c** Intermediate spot size on an *S/+* PO N_2_. Images were acquired by the authors

We sought to perform linkage analysis to identify genetic loci associated with *Dominant spot* by crossing BW *S/+* (Fig. 4a) with the PO laboratory stock of *P. polionotus* (+/+). F_1_ hybrids of *P. maniculatus* and *P. polionotus* exhibit developmental dysgenesis [1, 41]. When female *P. maniculatus* are crossed with male *P. polionotus*, the hybrid offspring are smaller than either parent, but are viable and fertile. In contrast, female *P. polionotus* crossed with male *P. maniculatus* result in overgrown fetuses with developmental defects and are not viable. Therefore, a male *+/+* PO was crossed with *S/+* BW to generate six F_1_ hybrids with forehead spots (Fig. 4b). These six *S/+* offspring were then backcrossed to PO *+/+* to generate an N_2_ generation containing 125 animals, of which 46 have spots (*S/+*) and 79 did not (*+/+*) (Fig. 4c).

**Fig. 5:**
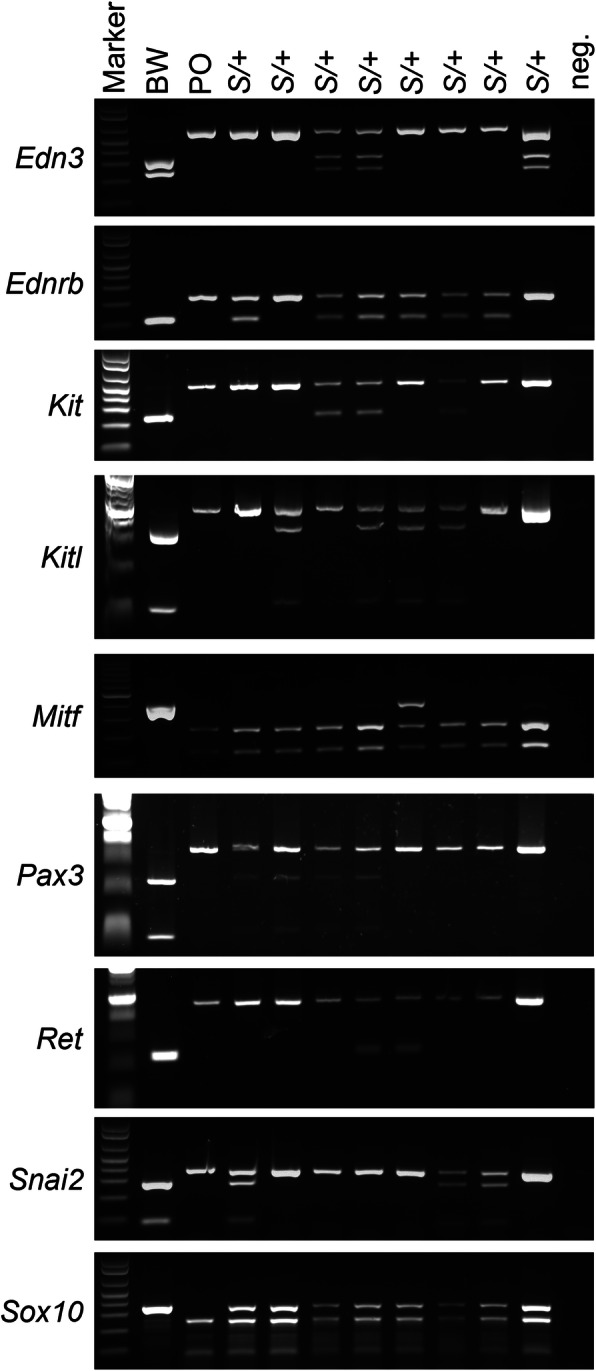
Linkage analysis for spotting mutation candidate genes. Genomic DNA from BW and PO control animals, along with a selection of *S/+* F_1_ animals, was PCR amplified for a set of candidate genes (*Edn3, Ednrb, Kit, Kitl, Mitf, Pax3, Ret, Snai2*, and *Sox10*), known to cause pigmentation defects in *Mus* and humans. Each amplicon contained a restriction enzyme recognition site polymorphism between BW and PO (see Table 5). PCR amplicons were digested with the appropriate restriction enzyme and analyzed by agarose gel electrophoresis. *Sox10* is the only candidate gene were all *S/+* N_2_ animals have both a PO and BW allele, demonstrating linkage with the *Dominant spot* phenotype. Each image was cropped to remove excessive space

Disrupted pigmentation patterns in laboratory mice, *Mus musculus*, are readily identifiable, and characterization of the causative mutations for these spotting defects has identified key members of a neural crest gene regulatory network necessary for normal neural crest development [42]. We pursued a candidate gene approach as a first step towards linking *Dominant spot* with a specific genomic region. *Edn3, Ednrb, Kit, Kitl, Mitf, Pax3, Ret, Snail,* and *Sox10* are all known to cause spotting phenotypes in *M. musculus*; therefore, we sought to identify allelic differences in *P. maniculatus* and *P. polionotus* for each gene to determine if any of these candidate genes are linked with *Dominant spot*. For our list of candidate genes, we identified a sequence variant that removes a restriction enzyme recognition site in one *Peromyscus* species. We will call these sites restriction fragment length polymorphisms (RFLPs) because of their similarity to the technique used for genomic variation analysis (Table 5). We then designed polymerase chain reaction (PCR) primers flanking the site to generate an RFLP site specific amplicon. BW and PO genomic DNA, along with genomic DNA from *S/+* N_2_ animals was PCR amplified and then digested with the appropriate restriction enzyme (Fig. 5 and Additional File 3). The *S* mutation arose in *P. maniculatus* and has been maintained on the BW stock; therefore, the *S* mutation occurs in a BW allele. If a candidate gene is linked with the *Dominant spot* trait, then all *S/+* N_2_ animals will have both a BW allele and a PO allele for that candidate gene. If an *S/+* N_2_ animal has only PO alleles for a candidate gene, then that candidate gene is not linked with the *Dominant spot* trait. From our list of candidate genes, eight of the candidate genes are not linked with *Dominant spot* as there are multiple *S/+* N_2_ individuals with only the PO allele. However, *Sox10* is linked with *Dominant spot*; 46 *S/+* N_2_ individuals were genotyped at the *Sox10* RFLP site, and all 46 are BW/PO (*χ*^2^ (1, *N* = 46) = 46, *p* = 1.2 X 10^− 11^). By employing the same RFLP analysis, we have identified a 1.7 Mb region between *Tex33* and *Pdgfb* on chromosome 20 that is linked with *Dominant spot* (Additional File 4). Among the 53 genes contained in the linkage interval, only *Sox10* has a defined role in neural crest development. Therefore, we favor the possibility that the *S* mutation disrupts *Sox10* function. We have sequenced the *Sox10* exons, exon/intron junctions, promoter, and several conserved enhancer regions but have not identified a sequence variation that disrupts *Sox10* function. We are expanding the sequencing analysis to include the entire linked region.

**Table 5:** Polymorphisms that generate RFLPs between BW and PO in candidate genes that cause spotting in *Mus*

Candidate Gene	Contig	PCR Amplicon Location	Polymorphism Location	BW sequence	PO Sequence	Forward Primer	Reverse Primer
*Edn3*	NW_006501107	1,645,861. .. 1,646,386	1,646,160	AGGCCT (StuI)	AGTCCT	CTCGAGAACCTTGGGATTCA	AACAGGGTCTCCTGCAGTGT
Ednrb*	NW_006501134	1,664,283. .. 1,664,495	1,664,389	CCGG (MspI)	CCAG	ATGACGCCACCCACTAAGAC	GATGATGCCTAGCACGAACA
*Kit*	NW_006501162	6,362,706. .. 6,363,108	6,362,907. .. 6,362,919	CCGT**GGTACC**TCTGCTCGGGA (KpnI)	CCGT//GGGA	CCCGTCCTAGCTTTGGAAC	AGCATCAGGGCAACCTTAAA
*Kitl*	NW_006501158	227,546. .. 228,022	227,668	GGATCC (BamHI)	GAATCC	CCCAATTAGCTGCTCTTCAAAC	GGAGCCTTTGTGTCTTATCAGTA
*Mitf*	NW_006501059	77,663,262. .. 7,663,281	7,663,508	GTATGC	GCATGC (SphI)	GGATGAGACTCAGGGTGAGG	GCTCCATCACTCGGCATTAT
*Pax3*	NW_006501055	3,517,274.. . 3,517,631	3,517,515	TTTAAA (DraI)	TTTCAA	CCTTGCCTACTACGCTCTGA	TAATTCTGCATCCTTCCGGC
*Ret*	NW_006501668	491,420. .. 491,911	491,673	AGGCCT (StuI)	AGACCT	GTTTCACCCTAGGAAGTTGTGG	GCCTCAGAAGCAGCCCTC
*Snai2*	NW_006502260	133,647. .. 133,992	133,912	TTTAAA (DraI)	TTTAAG	CCAAAGTTGAAGGCTGTTGC	AGTCCATTGCTTTCACACCT
*Sox10*	NW_006501150	898,573. .. 898,893	898,801	CCAC	CCGC (AciI)	GGCAGACTGAGGGAGGTGTA	GGAGATCAGCCACGAGGTAA

* Polymorphism and primers identified by and verified in our analysis

**Fig. 6:**
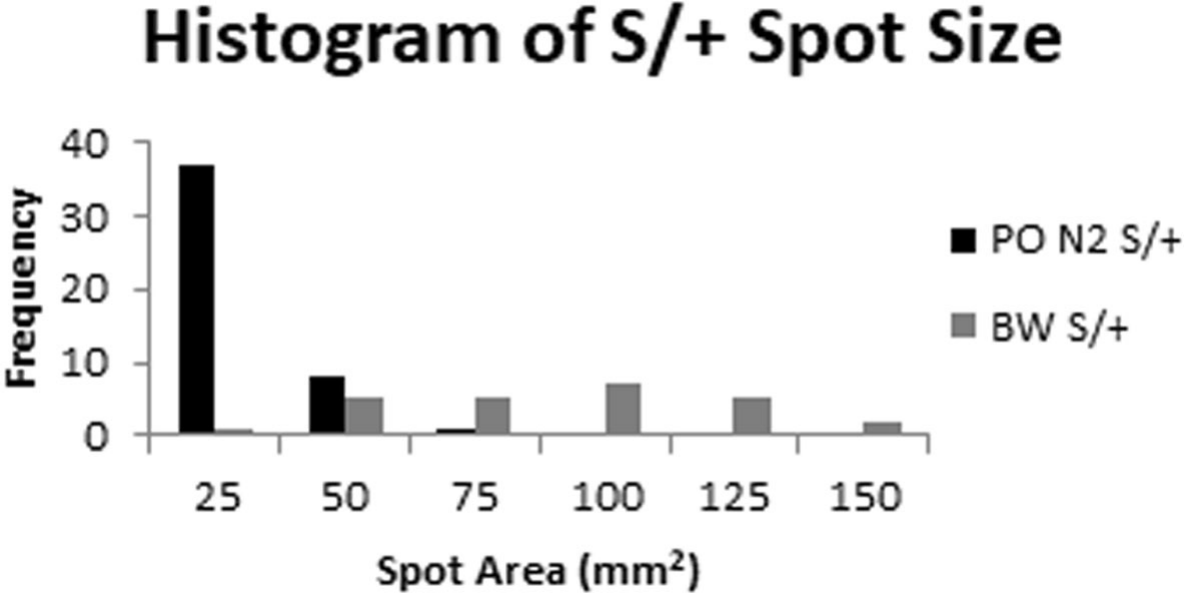
Histogram of spot size variation. This histogram displays the frequency of spot size area (mm^2^) for 25 BW *S/+* (gray) and 46 *S/+* on PO N_2_ (black)

In generating the N_2_ generation, we noticed that the spot size was smaller on the F_1_ and N_2_ animals compared to the originating BW background. The average spot size for 25 *S/+* BW animals is 77.6 ± 36.6 mm^2^. We crossed one BW *S/+* with PO *+/+* and generated six F_1_ *S/+* animals, which had very small spots in comparison, 3.75 ± 1.56 mm^2^, suggesting that PO alleles have a dominant effect on the *S/+* spot size phenotype. The six F_1_ *S/+* were backcrossed with PO *+/+* and in the PO N_2_ generation the spot size for 46 affected animals averaged 14.5 ± 13 mm^2^, which is significantly smaller than the spot size of *S/+* BW animals (Welch’s t [27] = 8.34, *p* = 5.38 × 10^− 9^), suggesting that genetic background has a significant impact on the *S/+* phenotype. A histogram for spot size for *S/+* on BW and PO N_2_ illustrates the quantitative nature of the phenotype and the shift in spot size in the PO N_2_ animals (Fig. 6). We used the backcross data to estimate the number of loci that affect spot size. The six F_1_ animals with small spots produced 46 offspring with spots, of which 17 (37%) resembled the F_1_ parent. In this backcross experiment there are only two possible genotypes for any gene. If one unlinked locus determines the spot size phenotype, then there are two possible genotypes that interact with *Dominant spot* and 50% of the offspring are expected to resemble the F_1_ parent. If two unlinked loci determine the spot size phenotype, then 25% should resemble the parent, and if three unlinked loci are involved then 12.5% should resemble the parent. Using a *X*^2^ analysis, we can reject a model for three interacting loci (*X*^2^ (1, *N* = 46) = 23.19, *p* = 1.47 X 10^− 6^), but not models for one interacting locus (*X*^2^ = (1, N = 46) = 3.13, *p* = 0.077) or two interacting loci (*X*^2^ (1, N = 46) = 2.82, *p* = 0.093). These data suggest that there are one to two modifiers that cause the observed variability in the *Dominant spot* trait. Discriminating between these two possibilities will require a larger sample size.

Further analysis indicates that there is a significant loss of affected animals in the N_2_ backcross. A total of 125 offspring were produced in the N_2_ generation. Of these animals, 46 pups had forehead spots, representing the *S/+* genotype, while 63 are expected (*X*^*2*^ (1, *N* = 125) = 9.25, *p* = 0.0024). Analysis of the PGSC breeding records for BW *S/+* X BW *+/+* indicate that the *S/+* genotype is produced at the expected frequency (310 total offspring of which 165 have spots, (*χ2* (1, *N* = 310) = 1.29, *p* = 0.256)). These results suggest that there is a significant loss of the *S/+* phenotype in the PO N_2_ offspring, resulting from either a lethality of *S/+* in PO N_2_ animals or because some PO N_2_ *S/+* animals have a phenotypic rescue and do not have a forehead spot. In the backcross, PO N_2_ animals without spots should have two PO alleles for *Sox10*, barring a rare recombination event that separates the *Sox10* RFLP site from the unknown causative mutation*.* We genotyped 50 PO N_2_ individuals without spots at the *Sox10* RFLP site and found that 21 are BW/PO, supporting the hypothesis that a modifier can phenotypically rescue the S/+ genotype. We are currently conducting a QTL analysis to identify loci associated with the spot size phenotype.

## Discussion

In 2001 Dewey and Dawson described *Peromyscus* as the “*Drosophila* of North American Mammalogy” [43]. At the time, the nearly completed *Mus* and human genome projects suggested that less traditional model systems would benefit from the application of the rich genetic resources provided by the completed *Mus* and human genomes. They believed that the increase in genomic resources would enable *Peromyscus* geneticists to identify the loci associated with specific behaviors and physiological traits associated with specific species or subspecies of *Peromyscus*. They were correct. The ability to generate fertile hybrids of *P. maniculatus* and *P. polionotus* coupled with QTL analysis has led to the identification of many loci associated with specific behaviors, such as burrow building and paternal parenting [15, 16]. The recent generation of genomic assemblies for *P. maniculatus*, *P. polionotus*, and *P. leucopus* should only quicken the pace of discovery [21].

To facilitate the use of *Peromyscus* for gene discovery, we have used available genomic resources to identify polymorphisms between *P. maniculatus* and *P. polionotus*. Our conservative estimate demonstrates that these closely related species have a polymorphism approximately every 68 base pairs. This genetic diversity supports the use these species for QTL analysis, as the sequence variation prevents large blocks of linkage disequilibrium, a characteristic that has been problematic for QTL analysis in *Mus* [4]. Most of the sequence variation occurs in intergenic or intronic regions; however, more than 10,000 genes have a polymorphism that results in a missense or nonsense variation between the two species, which could result in a functional difference between the *P. maniculatus* and *P. polionotus* alleles. GO term analysis on this list of proteins results in over 2000 GO terms that are overrepresented. This list is not necessarily informative based on the descriptive nature of GO terms at the top of the list, such as cellular or metabolic process, but it is more meaningful when used in combination with known physiological or behavioral differences between the species. *P. polionotus* have a 2-fold increase in blood triglycerides compared to *P. maniculatus* [24], and there are 62 genes associated with GO terms related to triglyceride metabolism. This selection of triglyceride associated genes provides a potential candidate gene list for identifying genetic variations associated with varying triglyceride levels. All these genes have human homologues, suggesting that variations identified in *Peromyscus* may inform human genetic variability associated with triglyceride levels.

The list of triglycerides associated candidate genes is a conservative estimate based on the possibility of protein functional changes. The number of genes is likely much higher, as changes in regulatory regions that affect transcriptional levels are also possible. We demonstrated the potential for polymorphisms affecting transcription by analyzing the CNS for ASH1L, an autism candidate gene [34]. We show that non-coding polymorphisms change predicted transcription factor binding sites for NKX2–1 and EBF1 in the ASH1L CNS. Mutations in NKX2–1 cause benign hereditary chorea and have recently been associated with autism [44]. *Nkx2–1* is expressed in the medial ganglionic eminence (MGE) and is critical for the production of inhibitory gamma amino butyric acid (GABA)eric cortical interneurons [45]. Reductions in these inhibitory neurons are associated with autism [46]. Common genetic variations in EBF1 have also been associated with premature birth and autism [47]. *Ebf1* is expressed in the lateral ganglionic eminence (LGE), which generates striatal projection neurons, including medium spiny neurons that are preferential lost in Huntington’s disease [48, 49]. *Ebf1* is essential for striatum formation and the generation of direct striatal projection neurons that project to the substantia nigra [50, 51]. Functional experiments are necessary to determine the potential impact of this and other non-coding polymorphisms on ASH1L transcription. Our comparative approach for both protein and DNA conservation demonstrates that the variations between BW and PO provides a rich source for functional analysis. Future QTL analysis between *P. maniculatus* and *P. polionotus* may help to narrow these candidate gene lists to identify genes that underlie the phenotypic difference between these species. Doing so will provide an animal model that will correlate genotypic variability with autism like behaviors. In addition, *Peromyscus* is an excellent model system for testing of future therapeutics, as the outbred nature of the stock will better model the variability of the human population than inbred *Mus* lines.

We combined linkage analysis, using *P. maniculatus* and *P. polionotus*, the polymorphisms between the two species, and a candidate gene approach to link the *Dominant spot* trait with *Sox10*. Mutations in *Sox10* are known to cause belly spotting and megacolon in *Mus* and Waardenburg syndrome, types 2E and 4C in humans [52–54]. Waardenburg syndrome is genetically heterogeneous and results from mutations in *Sox10, Pax3*, *Mitf*, *Snai2*, *Ednrb*, and *Edn3*, as well as many cases with unidentified mutations [55]. The phenotypic presentation of Waardenburg syndrome is variable, even in families with the same mutation, suggesting that there are genetic modifiers that affect the severity of the phenotype [56–58]. We see similar phenotypic variability in *Dominant spot*. *P. maniculatus* with the *Dominant spot* trait have a variable forehead spot size, tending towards larger spots. *Dominant spot* on the PO N_2_ background have significantly smaller spots. We believe that there are likely one to two genetic modifiers that interact with *Dominant spot* to cause the variability in spot size. Intriguingly, the *Dominant spot* trait is underrepresented on the PO N_2_ background, and likely results from a gene interaction that rescues the *Dominant spot* phenotype. A QTL analysis to identify loci associated with spot size variability is underway and may identify novel members of the neural crest developmental gene regulatory network (GRN) or novel alleles of known members of the GRN, which could expand our understanding of neural crest and melanocyte development. In addition to *Dominant spot*, the PGSC maintains the *Variable white* and *Tan streak* stocks, which also have pigmentation defects that are likely caused by mutations in the neural crest GRN [59, 60]. The improved genomic resources for *Peromyscus* combined with the three stocks of *P. maniculatus* with pigmentation defects and the inherent variability found in these outbred stocks makes *Peromyscus* an excellent model system for studying neural crest biology.

## Conclusions

The availability of genomic resources for *P. maniculatus* and *P. polionotus* facilitates the use of these rodent species to identify genomic loci associated with quantitative traits. We have conducted a linkage analysis for the *Dominant spot* trait, which arose in *P. maniculatus* by crossing it with *P. polionotus*. Using available genomic information, we identified RFLP polymorphisms between the two species in a set of candidate genes associated with pigmentation disruptions in *Mus* and humans and determined that a region of chromosome 20 containing *Sox10* is linked with the *Dominant spot* trait. Further analysis of the available genomic data demonstrates that there is significant genetic complexity between *P. maniculatus* and *P. polionotus* that underlies both physiological and behavior differences, including blood chemistry and stereotypic behaviors. QTL analysis for these traits will provide correlated loci that will aid in the identification of functional polymorphisms that generate phenotypic differences.

## Methods

### Polymorphism analysis

The first genomic assembly of the *P. maniculatus* genome, National Center for Biotechnology Information (NCBI) accession number GCF_000500345.1, was locally downloaded and used as the reference genome. Whole genome sequence reads, NCBI accession numbers SRX179420, SRX179421, and SRX179422, for *P. polionotus* were downloaded from the SRA database. After these sequences were converted into FASTQ format using the SRA Tool Kit (v. 2.1.16 centOS Linux 64-bit), the sequence reads, SRX179420 and SRX179422, which contained paired-end sequences reads, were split into two files, using Python script. The forward and reverse paired-end reads were linked to each other and the indexed adapter sequences were trimmed, using the Solexa QA package v. 1.13 [61]. End sequences were trimmed when the Phred quality score dropped below Q = 20. Trimmed PO sequence reads were aligned to the reference genome sequence, using the Burrows-Wheeler Aligner (BWA) program (v. 0.6.1-r104) [62]. The BWA default values for mapping were used, except that seed length (−l) was set to 28 and maximum differences in the seed (−k) equaled 1. Following alignment to the reference genome, data from each *P. polionotus* dataset was merged into one file. Sequence polymorphisms between the aligned *P. polionotus* sequence reads and the *P. maniculatus* reference genome were identified using the pileup function in SAMtools utilities (v. 0.1.16) [63]. The minimum read depth was set to 10 and the consensus sequence was generated with filter command “-uf”. SnpEff was utilized, using default parameters, to identify possible functional consequences of the SNP/Indel variant list produced from BCFtools. Data files for the sequence polymorphisms and annotations are available as .vcf files at https://osf.io/4eypx/.

GO analysis was performed on the list of genes containing nonsynonymous changes, as determined above. This list was compared to the *Mus* GO term database using the R packages Gostats [64] to identify highly statistically significant over and underrepresented GO terms with a *p* < 0.001.

ASH1L protein alignments were performed using CLC Main Workbench 8 (Qiagen). Protein sequences for human (NCBI accession number NP_060959.2), dog (NCBI accession number XP_537251.2), cow (NCBI accession number NP_001179672.1), *Mus* (NCBI accession number NP_619620.3), and rat (NCBI accession number NP_001101159.1) were imported into CLC Main Workbench from NCBI. Predicted BW ASH1L protein sequence was generated from the *P. maniculatus* reference genome (NCBI accession number GCF_000500345.1 and GeneID 102,924,929) and the *P. polionotus* protein sequence was generated from the *P. maniculatus* sequence with predicted nonsynonymous substitutions based on our polymorphism analysis.

The VISTA plot was generated using the mVISTA tool on the VISTA Gateway, http://genome.lbl.gov/vista/index.shtml [35, 65]. Sequences and annotations were downloaded from Ensembl, www.ensembl.org: cow chromosome 3:15111790–15,300,435 (NCBI accession number GCF_002263795.1); dog chromosome 7:42049263–42,231,760 (NCBI accession number GCF_000002285.3); human chromosome 1:155319268–155,568,307 (NCBI accession number GCF_000001405.39); *Mus* chromosome 3:92474023–92,630,085 (NCBI accession number GCA_001632555.1); and rat chromosome 2:188243220–188,391,250 (NCBI accession number GCF_000001895.5). *Peromyscus* sequences were from *P. maniculatus* scaffold NW_006501110:3142128–3,299,387 (NCBI accession number GCF_000500345.1) and *P. polionotus* chromosome 6:67063888–67,238,298 (NCBI accession number GCA_003704135.2).

Alignment of the conserved non-coding sequences in intron 3 was performed in CLC Main Workbench 8 with the following sequences: *P. maniculatus* scaffold NW_006501110:3241913–3,242,512; cow chromosome 3:15170453–15,171,101; dog chromosome 7:42120548–42,121,199; human chromosome 1:155462242–155,462,894; *Mus* chromosome 3:92529139–92,529,730; *P. polionotus* chromosome 6:67134871–67,135,471; and rat chromosome 2:188298601–188,299,194. Transcription factor binding site analysis was conducted using PROMO, http://alggen.lsi.upc.es/, using *Mus* transcription factors and binding sites with the *P. maniculatus* sequence NW_006501110:3242286–3,242,313 and *P. polionotus* sequence 6:67135070–67,135,097 [37, 38].

For restriction enzyme recognition site frequency analysis, the number of restriction enzyme recognition sites and average fragment length for each enzyme was calculated using the R package DECIPHER v2.0 using the *P. maniculatus* genome assembly (NCBI accession number GCF_000500345.1) [66].

### Peromyscus

All animal research was approved by the UofSC Institutional Animal Care and Use Committee. All animals used in this study were euthanized by CO_2_ asphyxiation, which is an American Veterinary Medical Association approved methodology for euthanasia for small rodents. *Dominant spot P. maniculatus* (*S/+*) on the BW laboratory stock and PO laboratory stock *P. polionotus* were obtained from the PGSC, https://www.pgsc.cas.sc.edu, and housed in solid-bottom opaque plastic cages with a wire bar lid, which serves as a food hopper and water bottle holder, and a filter top. Animals were group housed by sex, with up to six animals per cage, and provided paper-product bedding and nesting material. *Peromyscus* were housed with a 16 to 8-h light to dark cycle and feed food and water ad libitum. Matings were between one male and one female and pups were weaned at postnatal day 24. To generate timed pregnancies, *Dominant spot* (S/+) BW females and males were paired together. The following morning phosphate buffered saline, pH 7.3 (PBS) was used for a vaginal lavage on the females, and the wash checked for the presence of sperm. The presence of sperm was used to indicate pregnancy and noon of the day the sperm was detected was designated as e0.5. Embryos were collected on e13.5 and e14.5, photographed, and fixed in 4% paraformaldehyde in PBS overnight at 4 C. After fixation, embryos were washed in PBS and dehydrated into 70% ethanol for storage at − 20 C.

Using *Mus* linkage analysis as a guide, genotyping 50 mice can demonstrate linkage within 2 centiMorgans with 95% confidence [67]. Therefore, we set a goal of generating approximately 50 *Dominant spot* animals in a backcross experiment. One *S/+* BW female was mated with a +/+ PO male to generate an F_1_ generation. Six F_1_ *S/+* offspring with forehead spots (the *Dominant spot* trait) were then backcrossed to *+/+* PO animals to generate a PO N_2_ generation with 125 individuals. Both male and female N_2_ offspring were euthanized at weaning, photographed to document the spotting phenotype, and tail snips taken for genotyping. Within the N_2_ generation 46 animals had the *Dominant spot* trait and 79 were wild type. Photographs of forehead spots of 25 *Dominant spot* BW and 46 *Dominant spot* PO N_2_ were analyzed to determine the area of white fur, using the Fiji distribution of ImageJ [68]. The total number of animals used for all experiments, including the three generations of the backcross experiment and the timed pregnancies was 143.

PCR genotyping was performed for a set of neural crest candidate genes, using primers listed in Table 5, in a 25 μl reaction with a 63 °C annealing temperature for 30 cycles, using Dream Taq and Dream Taq Green Buffer (Thermo Fisher Scientific). The PCR amplicon was designed to contain a restriction enzyme recognition site in either the BW or PO allele of the candidate gene (see below for restriction enzyme selection). 0.5 μl of the appropriate restriction enzyme was added directly to the PCR mix and incubated at 37 °C for at least 2 h, before agarose gel electrophoresis on a 1.5% agarose gel. Images of *Peromyscus*, embryos, and electrophoretic gels were adjusted for brightness across the entire image, cropped to remove excessive space, and labels added using Adobe Photoshop.

## Supplementary information

**Additional File 1 **Overrepresented GO terms for the list of genes containing a nonsynomomous substitution between *P. maniculatus* and *P. polionotus*.

**Additional File 2 **Underrepresented GO terms for the list of genes containing a nonsynomomous substitution between *P. maniculatus* and *P. polionotus*.

**Additional File 3.** Electrophoresis gel images before cropping used for Fig. 5.

**Additional File 4 **Electrophoresis gel images for linkage analysis of *Tex33* and *Pdgfb*.

## Data Availability

Most of the data generated or analyzed during this study are included in this published article and its supplementary information files. The remaining datasets generated and/or analyzed during the current study are available at the following sources: the polymorphisms between *P. maniculatus* and *P. polionotus* and their functional annotations are located on the Open Science Framework repository of the Center for Open Science, https://osf.io/4eypx/ and DOI: 10.17605/OSF.IO/4EYPX. The National Center for Biotechnology Information (NCBI), https://www.ncbi.nlm.nih.gov/, stores many sequences analyzed in this studying, including *P. maniculatus* reference genome, GCF_000500345.1, https://www.ncbi.nlm.nih.gov/assembly/GCA_000500345.1. The *P. polionotus* sequence reads are available in the Sequence Read Archive (SRA) at NCBI, https://www.ncbi.nlm.nih.gov/sra, specifically SRX179420, https://www.ncbi.nlm.nih.gov/sra/SRX179420[accn], SRX179421, https://www.ncbi.nlm.nih.gov/sra/SRX179421[accn], and SRX179422 https://www.ncbi.nlm.nih.gov/sra/SRX179422[accn]. ASHIL protein sequences are available at NCBI, including human NP_060959.2, https://www.ncbi.nlm.nih.gov/protein/NP_060959.2/, dog XP_537251.2, https://www.ncbi.nlm.nih.gov/protein/XP_537251.2, cow NP_001179672.1, https://www.ncbi.nlm.nih.gov/protein/NP_001179672.1, *Mus* NP_619620.3 https://www.ncbi.nlm.nih.gov/protein/NP_619620.3, rat NP_001101159.1 https://www.ncbi.nlm.nih.gov/protein/NP_001101159.1, and *P. maniculatus* https://www.ncbi.nlm.nih.gov/gene/?term=102924929%5BUID%5D. The sequences and annotations used for the VISTA plot are available from Ensembl, https://useast.ensembl.org/, including cow chromosome 3:15111790–15300435, https://useast.ensembl.org/Bos_taurus/Gene/Summary?db=core;g=ENSBTAG00000003954;r=3:15121790-15298435, dog chromosome 7:42049263–42231760, https://useast.ensembl.org/Canis_lupus_familiaris/Gene/Summary?db=core;g=ENSCAFG00000016944;r=7:42046569-42225456;t=ENSCAFT00000026816, human chromosome 1:155319268–155568307, https://useast.ensembl.org/Homo_sapiens/Gene/Summary?db=core;g=ENSG00000116539;r=1:155335268-155562807, *Mus* chromosome 3:92474023–92630085, https://useast.ensembl.org/Mus_musculus/Gene/Summary?db=core;g=ENSMUSG00000028053;r=3:88950622-89079375, and rat chromosome 2:188243220–188391250, https://useast.ensembl.org/Rattus_norvegicus/Gene/Summary?db=core;g=ENSRNOG00000020386;r=2:188253220-188389250. *Peromyscus* sequences for the VISTA plot are available at NCBI, including *P. maniculatus* scaffold NW_006501110:3142128–3299387, https://www.ncbi.nlm.nih.gov/nuccore/NW_006501110.1/, and *P. polionotus* chromosome 6:67063888–67238298, https://www.ncbi.nlm.nih.gov/nuccore/CM010908.1.

## Abbreviations

BW: Laboratory stock of *P. maniculatus*
BWA: Burrows-Wheeler Aligner
CNS: Conserved non-coding sequences
ddRADseq: Double digest RADseq
INDELs: Insertions or deletions
GABA: Gamma amino butyric acid
GO: Gene ontology
GRN: Gene regulatory network
GWAS: Genome wide association studies
NCBI: National Center for Biotechnology Information
LL: Laboratory stock of *P. leucopus*
LGE: Lateral ganglionic eminence
MGE: Medial ganglionic eminence
PCR: Polymerase chain reaction
PGSC: *Peromyscus* Genetic Stock Center
PO: Laboratory stock of *P. polionotus*
QTL: Quantitative trait loci
RADseq: Restriction site-associated DNA sequencing
RE: Restriction enzyme
RFLP: Restriction fragment length polymorphisms
SFARI: Simons Foundation Autism Research Initiative
SI: Similarity index
SNP: Single nucleotide polymorphisms
SRA: Sequencing Read Archive
UofSC: University of South Carolina
UTR: Untranslated region
