# Supplementary figures and images for: Using genomic resources for linkage analysis in Peromyscus with an application for characterizing Dominant Spot
Source: BMC Genomics. 2020 Sep 11;21:622. doi: 10.1186/s12864-020-06969-1 (PMC7488232; doi:10.1186/s12864-020-06969-1)

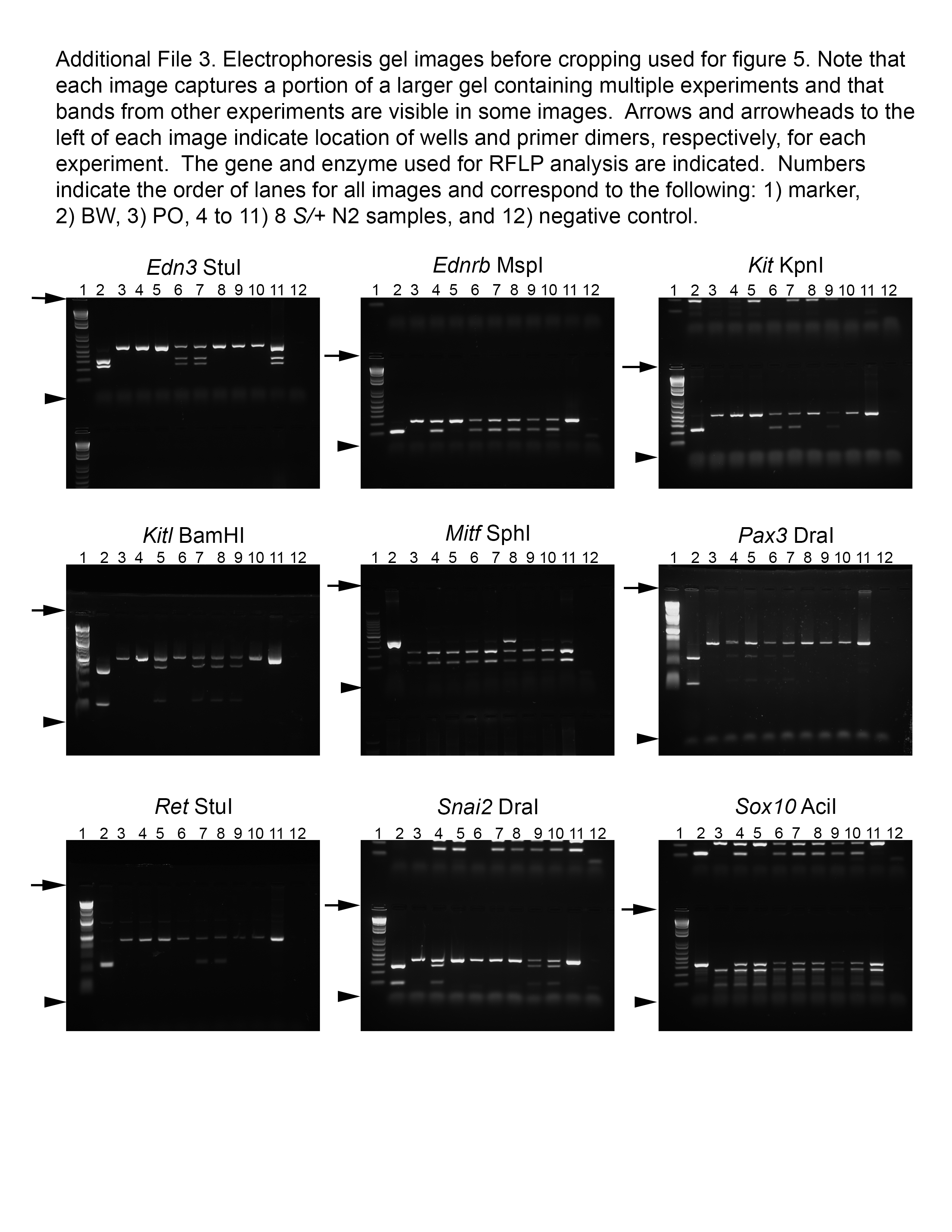

Supplement: Supplementary file 3 — Additional File 3. Electrophoresis gel images before cropping used for Fig. 5. [file 12864_2020_6969_MOESM3_ESM.tif]

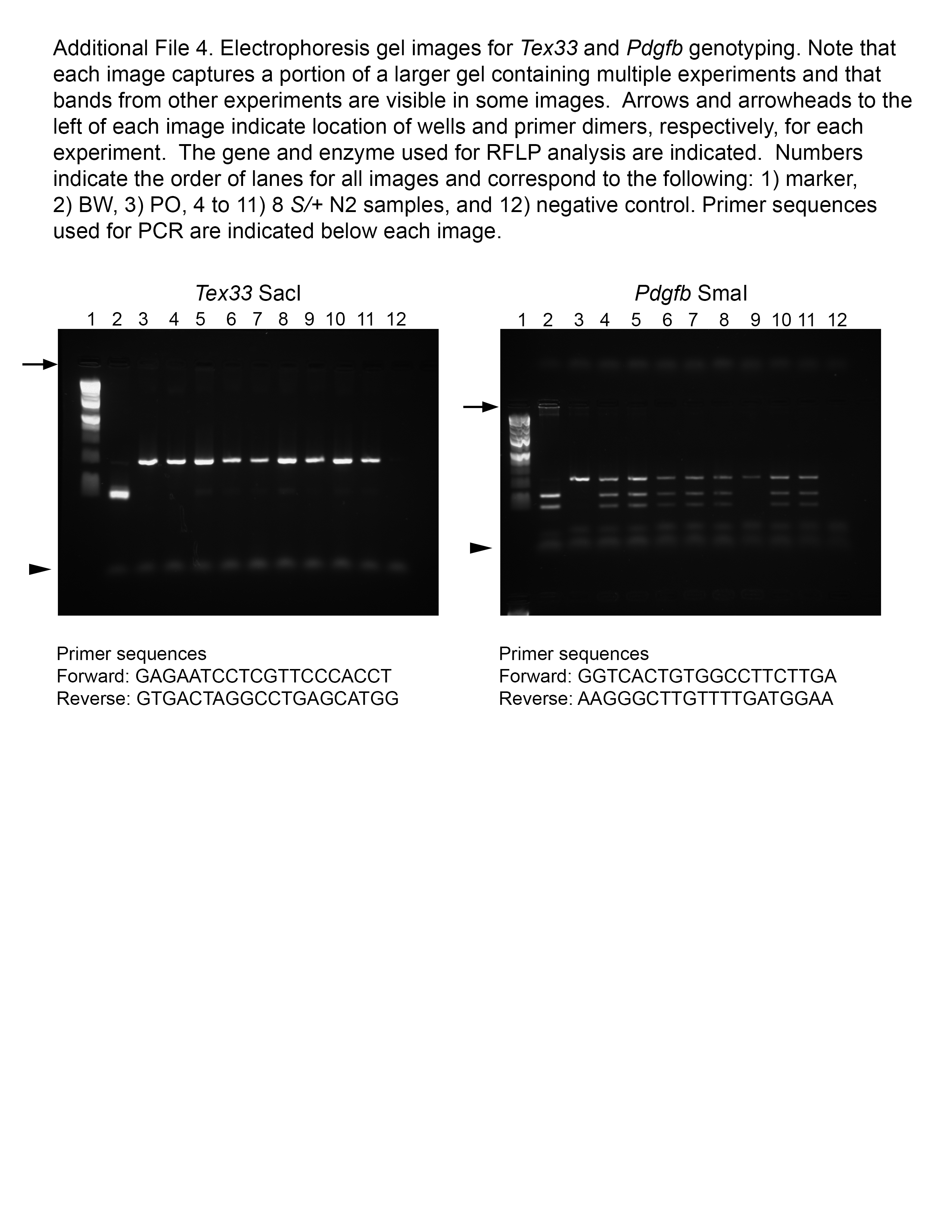

Supplement: Supplementary file 4 — Additional File 4 Electrophoresis gel images for linkage analysis of Tex33 and Pdgfb. [file 12864_2020_6969_MOESM4_ESM.tif]
